# Correction to ‘Characterization of an interplay between a *Mycobacterium tuberculosis* MazF homolog, Rv1495 and its sole DNA topoisomerase I’

**DOI:** 10.1093/nar/gkae1226

**Published:** 2024-12-04

This is a correction to: Feng Huang, Zheng-Guo He, Characterization of an interplay between a *Mycobacterium tuberculosis* MazF homolog, Rv1495 and its sole DNA topoisomerase I, *Nucleic Acids Research*, Volume 38, Issue 22, 1 December 2010, Pages 8219–8230, https://doi.org/10.1093/nar/gkq737.

In the original publication of this article, the blot in Figure 2B has accidentally been duplicated in Figure 6C (Rv1495-N(29-56). The error occurred during figure assembly.

The authors have provided the original raw data and a new Figure 6C, shown below.

This correction does not affect the results, discussion and conclusions presented in the article. These details have been corrected only in this correction notice to preserve the published version of record.


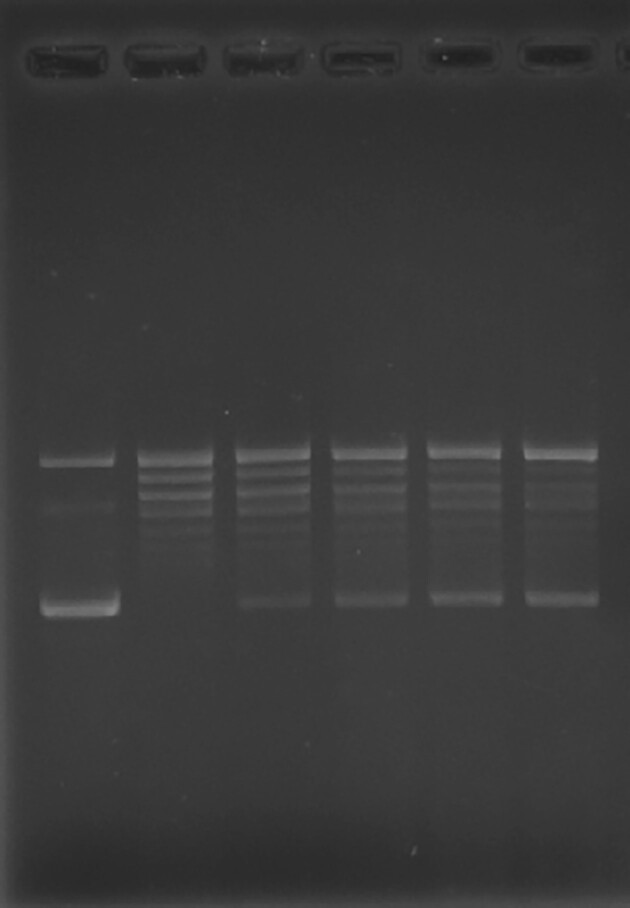


Original data Figure 2B


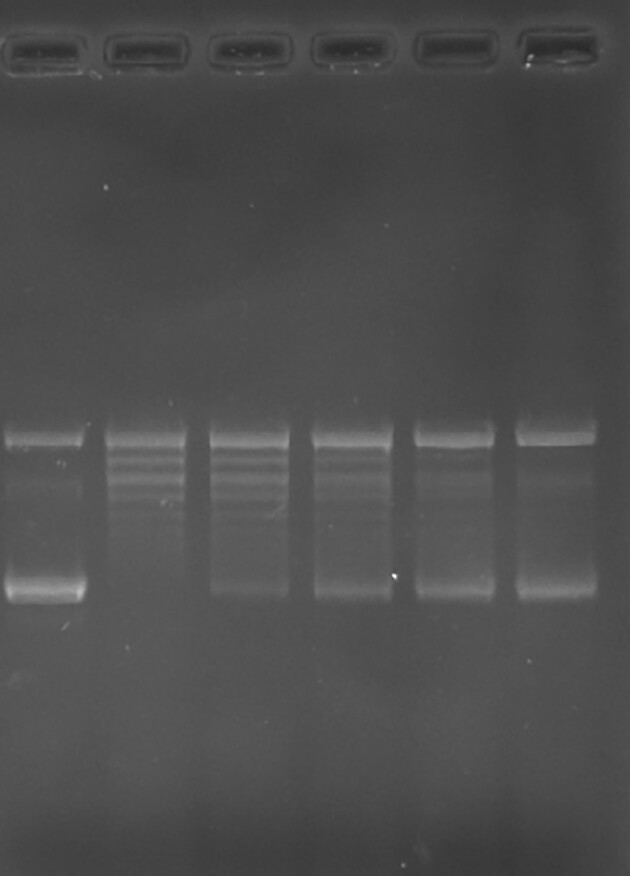


Original data Figure 6C


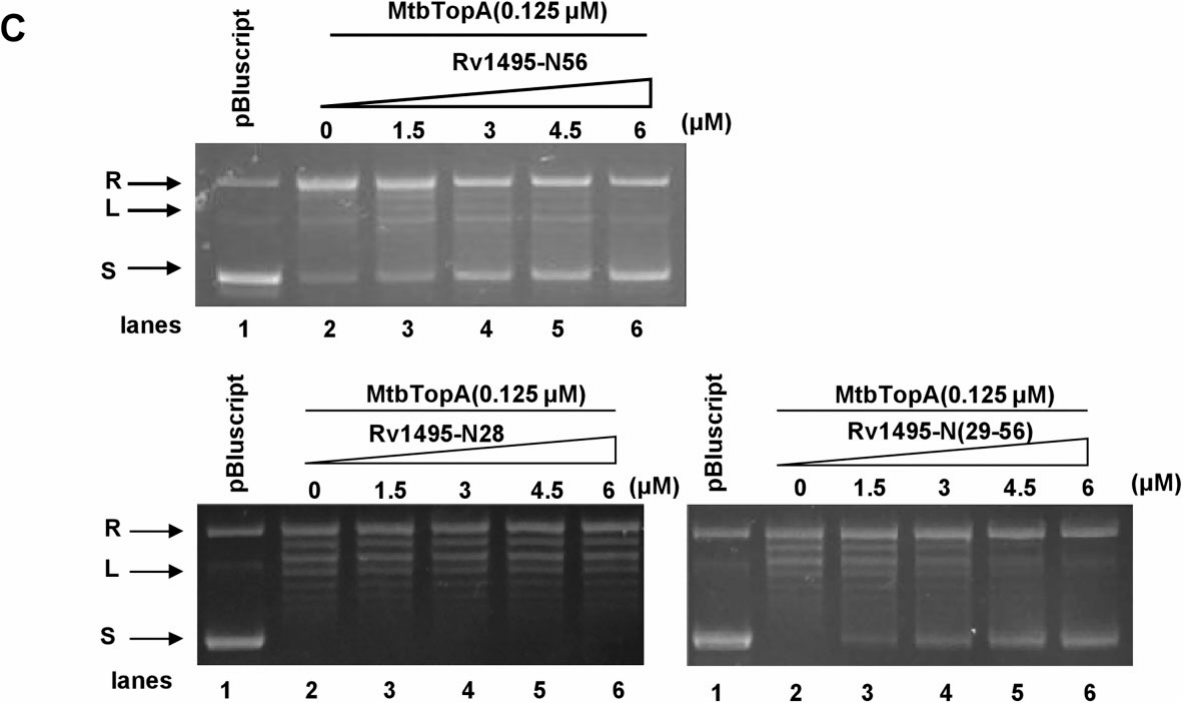


**Corrected Figure 6C**: Effect of different amount of Rv1495-N28, Rv1495-N56, and Rv1495-N(29-56) (0–6.0 μM) on topoisomerase activity.

